# Improving the accuracy of Respiratory Syncytial Virus (RSV) incidence estimates among hospitalised adults in Bristol, UK

**DOI:** 10.1186/s12879-025-11292-9

**Published:** 2025-08-21

**Authors:** Katie Lihou, Robert Challen, Anastasia Chatzilena, George Qian, Glenda Oben, Jade King, Serena McGuinness, Begonia Morales-Aza, Kaltun Duale, Ainhoa Rodriguez Pereira, William Healy, Jennifer Oliver, Nick Maskell, Adam Finn, Leon Danon, Catherine Hyams, Avon C. A. P. Research Group, Jade King, Serena McGuinness, Begonia Morales-Aza, Kaltun Duale, Ainhoa Rodriguez Pereira, William Healy, Aaran Sinclair, Amelia Langdon, Amy Taylor, Anabella Turner, Anna Jones, Anna Koi, Anya Mattocks, Bethany Osborne, Brianna Dooley, Callum Hawkins, Charli Grimes, Chloe Farren, Christian Povey, Claire Mitchell, David Adegbite, Dylan Thomas, Elinor Balch, Ella Ackroyd-Weldon, Emma Bridgeman, Emma Scott, Felix Wright, Ffion Davies, Fiona Perkins, Francesca Bayley, Gabriella Ruffino, Gabriella Valentine, Georgina Mortimer, Grace Tilzey, Harriet Ibbotson, Hanah Batholomew, Hugo Swift, Jacob Symanowski, Ilana Kelland, Imogen Ely, Jake Whittle, Jane Kinney, Johanna Kellett Wright, Jonathan Vowles, Josephine Bonnici, Josh Anderson, Juan Garcia-Tello, Julia Brzezinska, Julie Cloake, Katarina Milutinovic, Kate Helliker, Katie Maughan, Kazminder Fox, Kellie Pettinger, Konstantina Minou, Kyla Chandler, Lana Ward, Leah Fleming, Leigh Morrison, Liberty Smith, Lily Smart, Lisa Grimmer, Louise Setter, Louise Wright, Lucy Grimwood, Maddalena Bellavia, Madeleine Clout, Maia Lyall, Malak Eghleilib, Marianne Vasquez, Maria Garcia Gonzalez, Mariella Ardeshir, Marta Mergulhao, Martina Chmelarova, Matthew Randell, Michael Booth, Milo Jeenes-Flanagan, Miriama Resutikova, Monika Chaulagain, Natalie Chang, Nefeli Tavira, Nellie Farhoudi, Niall Grace, Nicola Manning, Oliver Griffiths, Olivia Pearce, Pip Croxford, Peter Sequenza, Petronela Anchidin, Rajeka Lazarus, Rebecca Clemence, Rosa Aldridge, Rhian Walters, Riley Cooper, Robin Marlow, Robyn Heath, Rupert Antico, Sandi Nammuni Arachchge, Sarah Stollery, Seevakumar Suppiah, Sean Robinson, Siddiqa Uddin, Taslima Mona, Tawassal Riaz, Teagan Barrett, Tom Long, Tudor Dimofte, Yassin Ben Khoud, Vicki Mackay, Zahra Hashmi, Zandile Maseko, Zoe Taylor, Zsuzsa Szasz-Benczur, Zsolt Friedrich

**Affiliations:** 1 Bristol Vaccine Centre, Schools of Population Health Sciences & Cellular and Molecular Medicine, University of Bristol, St Michael’s Hill, Bristol, BS2 8AE UK; 2 Academic Respiratory Unit, University of Bristol, Bristol, UK; 3 Engineering Mathematics, University of Bristol, Bristol, UK; 4 Clinical Research and Imaging Centre, UHBW NHS Trust, Bristol, UK

**Keywords:** Pneumonia, Lower respiratory tract infection, Cardiac failure, RSV, Test error

## Abstract

**Background:**

The burden of Respiratory Syncytial Virus (RSV) infection in adults is of interest in the context of recently-licensed vaccines. However, burden estimates are affected by test error associated with the testing platform, and number and type of samples tested.

**Methods:**

We conducted a prospective cohort study of adults with acute lower respiratory tract disease (aLRTD) hospitalised in Bristol, UK, from April 2022–March 2023. RSV was detected by RT-PCR both by routine standard-of-care (SOC) testing, and by testing of additional nasopharyngeal swabs, saliva and sputum samples from a patient subset. Latent class analysis was used to quantify and adjust for test error rates, including effects of multiple testing. RSV test-positivity rates are reported, and after adjustment for test error, are used to calculate adult population incidence/1000 person-years.

**Results:**

6906/11445 aLRTD cases (60%) were tested and 251 were positive (3.6%; 251/6906). Test-positivity peaked in December (95%CI 7.9–12.7%). Among cases, 43% had pneumonia, 55% had non-pneumonic infection, 59% chronic respiratory disease exacerbations, and 16% heart failure. Test-positivity was highest in 75–84-year-olds, and 30-day mortality was highest in ≥ 75-year-olds (7.1%; 9/127). Due to low positivity-rates and imperfect specificity (0.98–1.00), test-positivity (3.6%) overestimated inferred true prevalence (2.3%). After adjustment for test error, we estimate overall adult population incidence/1000-person-years to be 0.33 (0.21–0.49), and 2.02 (1.10–3.06) in ≥ 75-year-olds.

**Conclusions:**

RSV contributes significantly to hospitalised adult aLRTD, particularly among the elderly. The implementation of effective RSV vaccines could reduce morbidity, mortality and associated costs of disease. Adult RSV burden accuracy is improved by adjustment for test characteristics due to the impact of imperfect specificity when positivity-rates are low, and this is particularly important for out-of-season estimates. Multiple samples can improve burden estimation accuracy only when tests have near-perfect specificity.

**Supplementary Information:**

The online version contains supplementary material available at 10.1186/s12879-025-11292-9.

## Introduction

Respiratory syncytial virus (RSV) is a recognised cause of paediatric illness [1] and its importance in adult disease, including exacerbation of underlying heart and lung disease, is increasingly recognised [2, 3]. RSV severity in adults varies widely, from mild symptoms to hospitalisation and even death, particularly in older or frailer individuals [4]. A recent meta-analysis in high-income countries, pre-pandemic, found the pooled hospitalisation rate for RSV-related illness in ≥ 65-year-olds/1000 population/year was 1.57, and 3.47, after simple adjustment for diagnostic test sensitivity [5]. New RSV vaccines have been approved to prevent respiratory disease in adults (two protein subunit vaccines and a messenger RNA vaccine), with more in development, and roll-out of a new vaccination programme for older adults (> 75-year-olds) and pregnant women began in the UK in September 2024 [6–8]. Therefore, it is crucial to have accurate adult RSV-related disease burden estimates to assess the potential impact and cost-effectiveness of RSV vaccine strategies and inform future public health policy.

Few studies report RSV burden following SARS-CoV-2 emergence [9]. Social distancing measures implemented to reduce COVID-19 [10–12] affected the circulation of other respiratory pathogens. No prospective UK studies have reported the disease burden of RSV ARI hospitalisations among adults. Three retrospective pre-pandemic time-series modelling studies estimated disease burden among British older adults by associating the variability in an RSV-indicator (i.e., RSV laboratory surveillance data) with outcomes that included RSV-related events (i.e., respiratory hospitalisations). These UK studies reported an average annual incidence of 1.56/1000 population in > 65y [13] and 2.65/1000 in those with high-risk medical conditions; an annual incidence of 2.51/1000 respiratory hospital admissions [≥ 75y; 2010–2017] and 0.71/1000 respiratory hospital admissions [65–74y] [14]; and, average annual incidences of 0.90/1000 respiratory hospital admissions [65-74y], 2.80/1000 respiratory hospital admissions [75-84y] and 6.0/1000 respiratory hospital admissions [≥ 85y] [15].

Published RSV-related incidence among adults has varied substantially, driven by differences in methodology, underlying RSV prevalence, and testing-rates and characteristics. RSV testing in adults is infrequent due lack of RSV-specific treatments [16]. Clinical suspicion for RSV-associated cardiopulmonary disease exacerbation tends to be low and, consequently, these patients are often not tested [3]. Additionally, routine testing-rates of ARI patients decreases with patient age [17]. Other under-ascertainment sources include using case definitions that exclude some RSV disease presentations (e.g. pneumonia only), reduced sensitivity of single specimen PCR testing among older adults [18, 19], sampling period misaligning with RSV season, and delays between illness onset and sampling [20].

Test error rates act as a source of potential over- or under-estimation of disease burden estimates. However, test error is more likely to result in overestimation of RSV-related hospitalisation burden in adults as population incidence is generally low [21]. In low prevalence populations, imperfect test specificity, the true negative rate, can have a larger impact on disease burden estimates than imperfect test sensitivity [22]. As the relative proportion of disease-negative individuals is high, false positive-rates (1-specificity) result in a high ratio of false positives to true positives. Therefore, the proportion of test-positives correctly identified (positive predictive values (PPV)), will be low relative to the test negatives correctly identified (negative predictive values (NPV) [23, 24]. This is particularly problematic in study designs implementing multiple testing in parallel and classifying any positive test result as a positive individual, since combined test specificity decreases with increasing numbers of tests [25].

Realised test error rates are impacted by test sensitivity and specificity, as well as variations in the testing process, such as test settings, sample quality and volume, and patient characteristics. Latent class analysis (LCA) quantifies relative test accuracy within a specific system, even in the absence of a gold-standard reference test, incorporating factors impacting the false positive and false negative rate, including test specificity and sensitivity [26–28]. LCA classifies the probability of individuals being disease positive or negative, which are unobserved latent classes, based on the results of multiple imperfect tests that are combined to form a composite reference standard. LCA can quantify the effect of multiple testing on disease diagnosis certainty, and this method has been used to quantify test accuracy in the absence of a gold standard reference test for multiple pathogens [28–33]. Therefore, LCA can be used to adjust for study-specific test characteristics to improve the accuracy of disease burden estimation.

This analysis reports RSV test-positivity rate, by subgroup and over time, among adults in two large hospitals serving the population of Bristol, UK, using a prospective, population-based active surveillance study. LCA is used to adjust for test error rates and multiple testing in estimations of true RSV prevalence and population incidence, an error source commonly ignored in disease burden estimations [34]. The primary objective is to determine the RSV disease burden in this population following SARS-CoV-2 emergence and report incidence and its associated uncertainty.

## Methods

### Study design

This prospective observational cohort study included adults admitted to two large university hospitals (Southmead, and Bristol Royal Infirmary) in Bristol, UK. Adults (≥ 18y) admitted to both hospitals from 1 st April 2022–31st March 2023, encompassing all acute secondary care in Bristol, were screened for study inclusion. This time-period was selected as it included the first winter season unaffected by COVID-19 pandemic-related measures. Inclusion and exclusion criteria were designed to capture all aLRTD (acute lower respiratory tract disease) cases as previously published [35].

Demographic and clinical data were collected from medical records systematically using REDCap [36]. We collected data on co-morbidities at admission, determining Charlson co-morbidity index (CCI [37];) and Rockwood clinical frailty score (score 5–9 indicating frailty [38];). Vaccination records were obtained from linked general practitioner (GP) records. All cause 30-day mortality numbers were obtained by linkage with the national healthcare records using a unique patient identifier.

### Respiratory specimen testing

Standard-of-care (SOC) virological testing results obtained from naso- or oropharyngeal (NP/OP) swabs were taken from patients’ medical charts. SOC samples underwent RSV testing by RT-PCR in the local UKHSA clinical microbiology lab using either Hologic Panther Fusion, BioFire Diagnostics system, or Cepheid Xpert® panel tests. Patients who consented to participate in the enhanced diagnostic testing study arm underwent research sampling between April 2022–March 2023, providing one or more of the following specimen types: an upper respiratory swab (NP or combined NP/OP swab), a saliva, or sputum sample. If subjects were unable to produce saliva, a saline mouth wash specimen was collected. Research specimens were tested by RT-qPCR using Certest Biotech Viasure® viral pathogen multiplex panels on the Applied Biosystems QuantStudio 7 Flex Real-Time PCR System (Thermo Fisher Scientific). The sample volumes, PCR threshold and baseline values used, differed between SOC and research RT-PCR tests. Research swabs were stored in STGG (skim-milk, tryptone, glucose, glycerine) medium and SOC swabs in VTM (viral transport medium).

### Case definitions

aLRTD was defined as any presentation with acute lower respiratory disease, including pneumonia; non-pneumonic-LRTI (NP-LRTI); acute bronchitis; exacerbations of underlying cardiorespiratory disease (CRDE) including chronic obstructive pulmonary disease (COPD) and asthma; and acute or decompensated heart failure (HF). Patients were designated RSV test-positive if they tested positive on PCR, from any specimen type. Test-positivity refers to the overall unadjusted test positivity in the population—the proportion of RSV-tested participants that had at least one positive test result for RSV, by any test. This is the equivalent to the observed, or the ‘apparent’ RSV prevalence in the tested population. When referring to a specific test, test-positivity indicates the proportion of participants tested by that method who tested positive by that method. True prevalence refers to the actual RSV burden in the population, which in this context is either simulated from synthetic data, or inferred from test-positivity and model adjustment, and is specified in either case. For full case definitions see: Extended Data, 1 [39, 40]).

### Statistical analysis

**Fig. 1:**
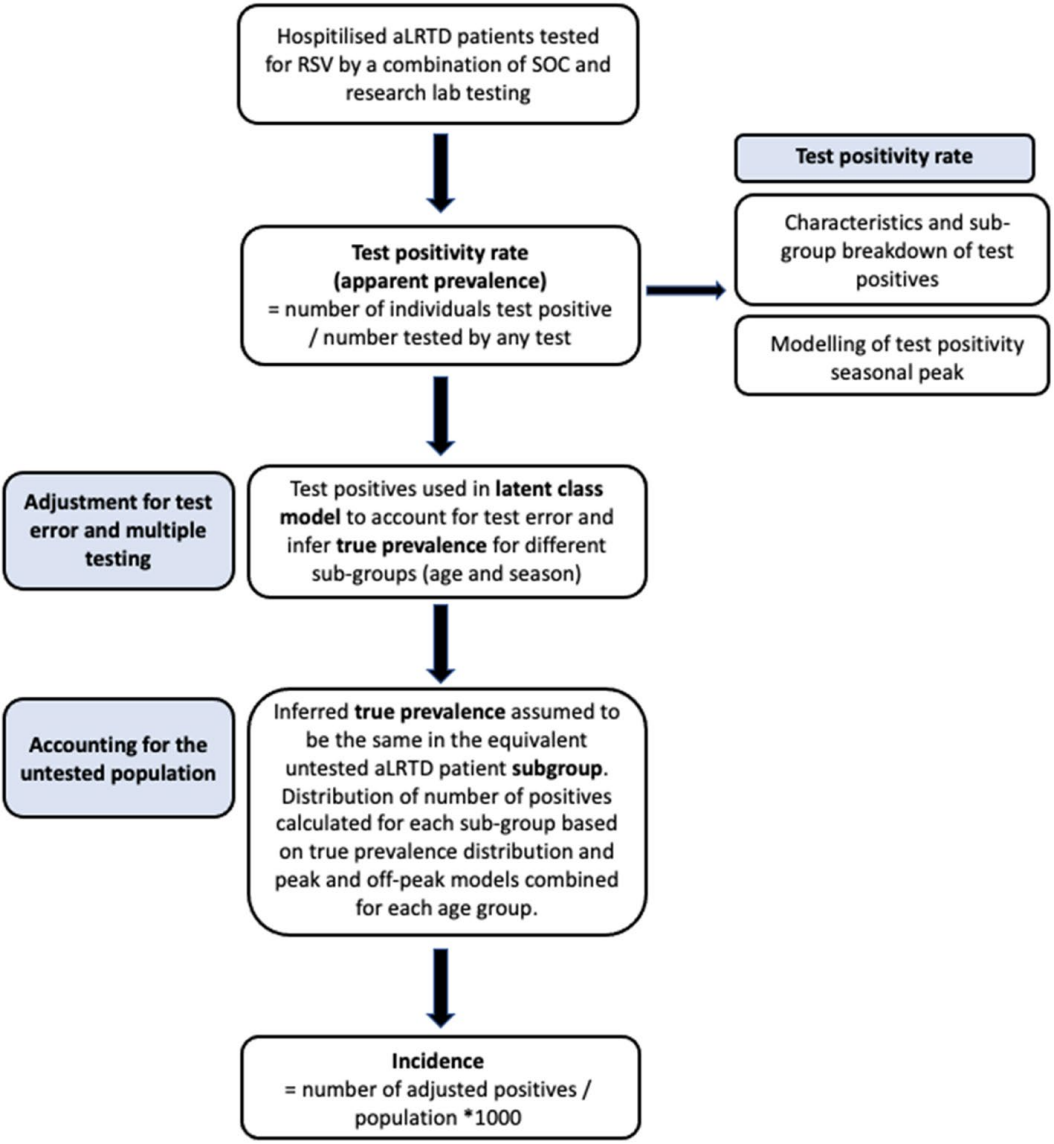
Flowchart of methods used to calculate annual RSV population incidence in adults in Bristol between April 2022 – March 2023

The primary objectives were to report RSV test-positivity rates among the RSV tested aLRTD hospitalised population and sub-categories, assuming any positive test represented an RSV-positive individual, and to estimate population incidence rates with appropriate uncertainty, whilst accounting for test error and multiple testing (Fig. 1). Comparisons between different participant populations were made using Kolmogorov–Smirnov tests for continuous variables, and Fisher’s exact test for categorical variables. Test-positivity rates were calculated as the number of participants RSV test-positive/100 aLRTD hospital admissions tested for RSV. Results are stratified by age and aLRTD subgroups. Apart from pneumonia and NP-LRTI, these subgroups are not mutually exclusive. RSV test-positive-rates over time were estimated using the weekly number of individuals that were RSV test-positive, across any test, and the weekly number of individuals tested for RSV in the aLTRD admitted population, fitted to a quasi-binomial model assuming a time-varying rate. Data were locally fitted with order 2 polynomial and a logit link function using the methods of [41]. All analyses were conducted using R [42].

### Latent class analysis (LCA)

Latent class analysis (LCA) was used to adjust test-positivity in the tested population for test error and multiple testing to infer true prevalence in the tested population. The LC model was built using a Bayesian framework and implemented in Stan using the No-U-Turn sampler variant of Hamiltonian Monte Carlo through the R interface Rstan [43]. The probability of an individual having a particular pattern of test outcomes is conditional on true disease prevalence and test sensitivity and specificity, which are unobserved latent parameters estimated by the model (for details see Supplementary). Sensitivity is the probability that the test will be positive if an individual is infected with RSV (Se = *P*(Test +| Infected)), and specificity is the probability that the test will be negative if an individual is not infected with RSV (Sp = *P*(Test—| Not infected)); the model requires no assumptions about these as the agreement and disagreement between multiple tests provides enough information to infer them. LC models assume all tests are conditionally independent of one another, therefore an individual varying random effect with a scaling parameter was included to model the conditional dependence between the three research sample types [30, 44, 45].

Model accuracy was confirmed using simulated data with known parameters of true prevalence, test sensitivity, and test specificity. Test error associated with multiple testing was quantified by combining individual inferred test sensitivities and specificities (see Supplementary).

### Population incidence

The LC model was run for different subgroups of the RSV-tested aLRTD population: the peak RSV season (Nov-Feb), the off-peak season and the peak season split by age group. Incidence was calculated in each age group by assuming the same inferred true prevalence in the tested and untested subgroup populations (Number of RSV-positives = inferred true prevalence * number of aLRTD hospitalised patients in subgroup). The number of positives in each age group was based on the median from the combined distributions of the number of positives from peak/off-peak models. Uncertainty in the number of positives was based on 95% credible intervals calculated using the package ‘bayestestR’ (method = HDI [46]). The population denominators were defined based on historical patient healthcare utilisation data for the two Bristol hospitals in the study to estimate the hospitals catchment population, as previously described [47]. These numbers were used to calculate the adult population RSV incidence/1000-person-years with appropriate uncertainty (Fig. 1).

## Results

### Characteristics of RSV testing

**Fig. 2:**
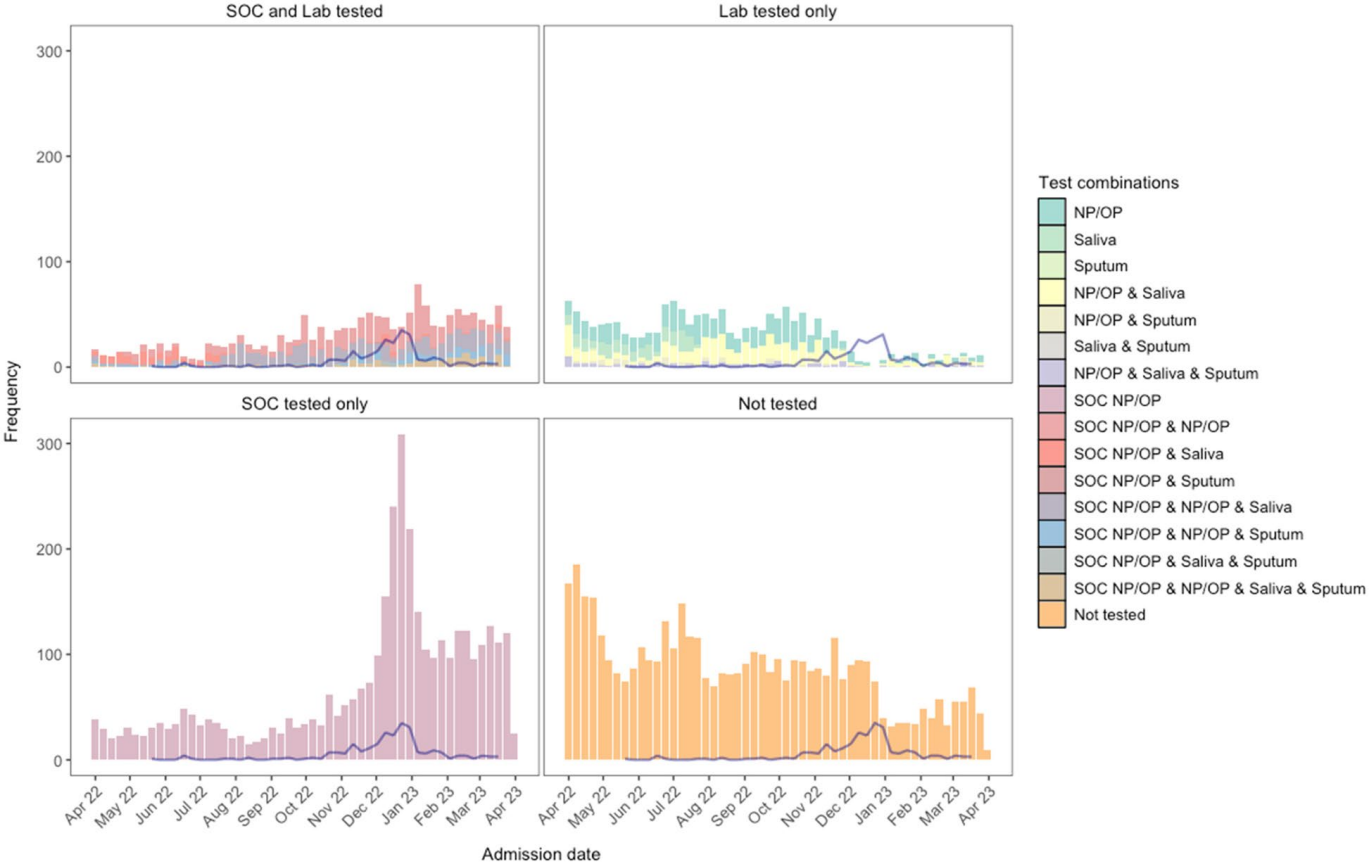
Test and sample type combinations for testing of RSV across the study time period. Histogram of specimen combinations collected from each study participant (between April 2022 – March 2023); SOC NP/OP swab; research NP/OP swab; research saliva sample; and research sputum sample. Weekly counts of RSV, by a positive on any test, are shown by the blue line

Between 1 st April 2022 and 31 st March 2023, 118,819 adults were admitted at the two study sites: 11,445 (10%) had aLRTD. 6,906 (60%) were tested for RSV (Extended Data, 2;3): 3,632 (53%) were tested by standard-of-care (SOC); 1,633 (24%) by research sampling; and 1,641 (24%) using both approaches (Extended Data, 4). Median time from admission to first SOC test was 0 d (days; IQR:0–2), and to research test was 2 d (IQR:1–3; Extended Data, 5). Testing-rate varied throughout the year, peaking over the winter due to increased SOC testing (Fig. 2). Overall, 39.7% (4539/11,445) of participants and during peak season (Nov-Feb), 26.2% (900/3,432) were untested. The median age of the tested participants was 73.4y (IQR:60.3–82.5), and this differed somewhat between participants by testing-type (Extended Data, 4), with the biggest difference in ≥ 85-year-olds: 23.1% of SOC-only-tested were ≥ 85y compared to 16.8% of research-only-tested. RSV-tested participants were slightly younger than untested participants (Extended Data, 3). Among those tested, 7.2% resided in care homes, the median CCI score was 4.0 (IQR:3.0–6.0), and 61.3% were smokers/ex-smokers. Tested participants were more likely to have pneumonia and CRDE than untested participants (47.0% versus 37.4%, and 51.2% versus 40.9%, respectively; Extended Data, 3). A full breakdown of RSV-tested participants by presenting symptom is presented in Extended Data, 6.

Of participants undergoing RSV testing, 4.4% (95%CI:3.9–5.0%; 232/5273) tested positive on SOC testing alone and 3.6% (3.2–4.1; 251/6906) through any test (i.e., SOC NP/OP; research NP/OP, saliva, or sputum): 232/251 (92.4%) RSV test-positive participants were identified through SOC testing alone (Table 1). The percentage of RSV test-positive individuals varied across individuals with different combinations of tests (Fig. 5). Of the research samples, the RSV test-positive-rate was 1.1% (0.7–1.5) for NP/OP, 0.9% (0.5–1.5) for saliva, and 3.4% (2.0–5.3) for sputum samples (Table 1).

**Table 1:** Test-positive-rates of the different RSV tests in the study. The number tested, percentage tested, number positive, and percentage positive of different types of test/samples for RSV in the study. 95% CIs are Clopper and Pearson

Study Specimens	n, tested	%, tested with specimen type	n, positive by specimen type	%, positive of sample tested	95% CI
Overall	6906	100.0%	251	3.6%	3.2–4.1%
SOC NP/OP swab	5273	76.4%	232	4.4%	3.9–5.0%
Research lab, any sample	3250	47.1%	47	1.4%	1.1–1.9%
NP/OP swab	2887	41.8%	31	1.1%	0.7–1.5%
Saliva^a^	1629	23.6%	15	0.9%	0.5–1.5%
Sputum	530	7.7%	18	3.4%	2.0–5.3%

^a^In subjects that were unable to produce saliva, a saline mouth wash was obtained (1.9%; 31/1629)

### Characteristics of RSV-positive participants

Of the 251 RSV test-positive participants, 109 (43%) had pneumonia, 137 (55%) NP-LRTI, 149 (59%) CRDE, and 40 (16%) HF. 4.2% (149/3535) of CRDE patients and 3.2% (40/1265) of HF patients, that were tested for RSV, tested positive for RSV. RSV test-positives were found in all age groups, including 18–34-year-olds, while 75–84-year-olds had the highest percentage of RSV test-positives (Table 2). All RSV test-positive patients aged 18-34y had comorbid disease (data not shown). Among RSV test-positive participants, 5 (2%) also had a positive COVID-19 test, 6 (2%) a positive influenza-A test, and 1 (0%) a positive influenza-B test. The median length of hospital admission was 5 d (IQR:2–8). Overall, 3.6% of RSV test-positive patients were admitted to ICU, remaining there for an average of 6 d (IQR:3–14). The all-cause 30-day mortality rate was 4.4% and was highest among ≥ 75-year-olds (7.1%), broadly increasing with patient age (Table 3).

**Table 2:** Patient characteristics of adults hospitalised with aLRTD that were test-positive for RSV by standard of care (SOC) or research lab. Patient demographics are shown for all adults hospitalised with aLRTD during the study period that had an RSV-positive test from any test (total, pneumonia, NP-LRTI, CRDE, HF). Full demographics by RSV testing are presented in Extended Data, 3;4

		**Confirmed RSV infection**									
		**Total**		**Pneumonia**		**NP-LRTI**		**CRDE**		**HF**	
**Variable**	**Characteristic**	**N**	**Value**	**N**	**Value**	**N**	**Value**	**N**	**Value**	**N**	**Value**
Age	Median (IQR)	251	75.0 (61.6–82.4)	109	77.5 (64.7–82.7)	137	73.2 (60.0–82.2)	149	73.1 (63.0–80.7)	40	79.9 (64.7–85.7)
CCI	Median (IQR)	251	4.0 (3.0–6.0)	109	4.0 (3.0–6.0)	137	4.0 (3.0–5.0)	149	4.0 (3.0–6.0)	40	5.22 (40)
Age category (y) ^a^	18–34	10	4.0%	4	3.7%	5	3.6%	6	4.0%	1	2.5%
	35–49	23	9.2%	5	4.6%	16	11.7%	12	8.1%	1	2.5%
	50–64	43	17.1%	19	17.4%	24	17.5%	25	16.8%	9	22.5%
	65–74	48	19.1%	21	19.3%	27	19.7%	38	25.5%	3	7.5%
	75–84	81	32.3%	39	35.8%	40	29.2%	50	33.6%	15	37.5%
	≥ 85	46	18.3%	21	19.3%	25	18.2%	18	12.1%	11	27.5%
Gender	Male	122	48.6%	52	47.7%	68	49.6%	67	45.0%	20	50.0%
	Female	129	51.4%	57	52.3%	69	50.4%	82	55.0%	20	50.0%
Pneumonia	yes	109	43.4%	109	100.0%	0	0.0%	65	43.6%	21	52.5%
NP-LRTI	yes	137	54.6%	0	0.0%	137	100.0%	79	53.0%	19	47.5%
CRDE	yes	149	59.4%	65	59.6%	79	57.7%	149	100.0%	23	57.5%
HF	yes	40	15.9%	21	19.3%	19	13.9%	23	15.4%	40	100.0%
Ethnicity	White British	183	72.9%	76	69.7%	102	74.5%	109	73.2%	28	70.0%
	White other	7	2.8%	4	3.7%	3	2.2%	5	3.4%	2	5.0%
	Black	3	1.2%	1	0.9%	2	1.5%	0	0.0%	0	0.0%
	Asian	5	2.0%	1	0.9%	4	2.9%	3	2.0%	0	0.0%
	Mixed origin	3	1.2%	0	0.0%	3	2.2%	0	0.0%	2	5.0%
	Other	2	0.8%	2	1.8%	0	0.0%	2	1.3%	0	0.0%
	Unknown	48	19.1%	25	22.9%	23	16.8%	30	20.1%	8	20.0%
Care home resident	no	232	92.4%	98	89.9%	129	94.2%	141	94.6%	37	92.5%
	Yes	19	7.6%	11	10.1%	8	5.8%	8	5.4%	3	7.5%
Smoker	Current	35	13.9%	17	15.6%	18	13.1%	24	16.1%	5	12.5%
	Ex-smoker	117	46.6%	52	47.7%	62	45.3%	77	51.7%	18	45.0%
	Non-smoker	78	31.1%	28	25.7%	49	35.8%	36	24.2%	13	32.5%
	Unknown	21	8.4%	12	11.0%	8	5.8%	12	8.1%	4	10.0%
COVID-19 vaccination	Not received	19	7.6%	4	3.7%	14	10.2%	9	6.0%	1	2.5%
	Received	232	92.4%	105	96.3%	123	89.8%	140	94.0%	39	97.5%
	Unknown	0	0.0%	0	0.0%	0	0.0%	0	0.0%	0	0.0%
CURB65^c^	Low	134	53.4%	44	40.4%	86	62.8%	81	54.4%	15	37.5%
	Moderate	87	34.7%	46	42.2%	40	29.2%	52	34.9%	16	40.0%
	Severe	30	12.0%	19	17.4%	11	8.0%	16	10.7%	9	22.5%
COPD	no	171	68.1%	68	62.4%	100	73.0%	69	46.3%	25	62.5%
	yes	80	31.9%	41	37.6%	37	27.0%	80	53.7%	15	37.5%
Asthma	no	184	73.3%	89	81.7%	93	67.9%	83	55.7%	33	82.5%
	yes	67	26.7%	20	18.3%	44	32.1%	66	44.3%	7	17.5%
Bronchiectasis	no	250	99.6%	109	100.0%	137	100.0%	148	99.3%	40	100.0%
	yes	1	0.4%	0	0.0%	0	0.0%	1	0.7%	0	0.0%
IHD	no	221	88.0%	97	89.0%	119	86.9%	132	88.6%	31	77.5%
	yes	30	12.0%	12	11.0%	18	13.1%	17	11.4%	9	22.5%
Hypertension^b^	no	219	87.3%	97	89.0%	117	85.4%	133	89.3%	36	90.0%
	yes	32	12.7%	12	11.0%	20	14.6%	16	10.7%	4	10.0%
On immunosuppression	no	199	79.3%	85	78.0%	111	81.0%	107	71.8%	36	90.0%
	yes	52	20.7%	24	22.0%	26	19.0%	42	28.2%	4	10.0%
Diabetes type	None	199	79.3%	88	80.7%	107	78.1%	116	77.9%	28	70.0%
	Type 1	3	1.2%	2	1.8%	0	0.0%	1	0.7%	0	0.0%
	Type 2	49	19.5%	19	17.4%	30	21.9%	32	21.5%	12	30.0%
CKD^d^	None	185	73.7%	78	71.6%	103	75.2%	113	75.8%	28	70.0%
	Mild	56	22.3%	24	22.0%	31	22.6%	30	20.1%	7	17.5%
	Moderate and severe	10	4.0%	7	6.4%	3	2.2%	6	4.0%	5	12.5%

*aLRTD*, acute lower respiratory tract disease, *CCI* Charlson comorbidity index, *CKD* Chronic kidney disease, *COPD* Chronic obstructive pulmonary disease, *CRDE* Chronic respiratory disease exacerbation, *HF* Heart failure, *IHD* Ischaemic heart disease, *NP-LRTI* Non-pneumonic lower respiratory tract infection, *PCR* Polymerase chain reaction, *RSV* Respiratory syncytial virus, *SD* Standard deviation

^a^In the UK, patients aged ≥ 65 years are eligible for Pneumococcal vaccination (PneumoVax®, PPV23) once, and annual influenza vaccine

^b^Hypertension was only included if causing other cardiac complications

^c^CURB-65 score 0–1 = low, 2 = moderate and ≥ 3 = severe

^d^Chronic kidney disease (CKD) was classified as mild if stage 1–3; moderate/severe if stage 4–5, end-stage renal failure or there was dialysis dependence

**Table 3:** Outcomes of adult patients hospitalised with aLTRD and test-positive for RSV. The median hospital length of stay, percentage intensive care or high-dependency unit (ICU/HDU) admission, median ICU/HDU length of stay, and percentage all-cause mortality within 30-days of admission among adults hospitalised with aLTRD and test-positive for RSV. These are reported as the percentage of patients test-positive for RSV among RSV tested patients

Variable	All included aLTRD	Tested for RSV	Test-positive for RSV				
	**Total**	**Total**	**Total**	**Pneumonia**	**NP-LRTI**	**CRDE**	**HF**
**Length of stay Median days (IQR)**							
Overall	5 (2–13)	6 (3–14)	5 (2–8)	6 (3–10)	5 (2–7)	5 (2–8)	8 (4–16)
18-64y	3 (1- 7)	4 (2- 9)	4 (2- 7)	4 (3–12)	3 (2–6)	4 (2- 6)	6 (4–30)
65-74y	5 (2–12)	6 (3–13)	5 (3- 7)	4 (2- 8)	5 (3–6)	4 (2- 6)	10 (8–10)
≥ 75y	7 (3–18)	8 (4–18)	6 (3–10)	6 (4–12)	5 (2–8)	6 (2–12)	8 (3–12)
**ICU/HDU admission N (%)**							
Overall	331/11445 (2.9%)	228/6906 (3.3%)	9/251 (3.6%)	7/109 (6.4%)	2/137 1.5%)	4/149 (2.7%)	3/40 (7.5%)
18–64	150/3705 (4%)	102/2224 (4.6%)	4/76 (5.3%)	3/28 (10.7%)	1/45 (2.2%)	1/43 (2.3%)	1/11 (9.1%)
65–74	92/2255 (4.1%)	69/1533 (4.5%)	0/48 (0%)	0/21 (0%)	0/27 (0%)	0/38 (0%)	0/3 (0%)
≥ 75y	89/5485 (1.6%)	57/3149 (1.8%)	5/127 (3.9%)	4/60 (6.7%)	1/65 (1.5%)	3/68 (4.4%)	2/26 (7.7%)
**ICU/HDU length of stay Median days (IQR)**							
Overall	6 (3–10)	6 (3–11)	6 (3–13)	6 (3–11)	13 (8–14)	10 (5–13)	3 (2–4)
18-64y	6 (3–12)	6 (3–12)	13 (6–14)	6 (4–18)	14 (13–14)	10 (8–11)	6 (6- 6)
65-74y	6 (3–10)	6 (3–11)	-	-	-	-	-
≥ 75y	6 (3–8)	6 (4–8)	4 (2- 7)	5 (3- 9)	3 (3- 3)	7 (4–10)	2 (2- 2)
**All-cause mortality N (%)**							
Overall	1000/11445 (8.7%)	494/6906 (7.2%)	11/251 (4.4%)	9/109 (8.3%)	2/137 (1.5%)	5/149 (3.4%)	6/40 (15%)
18-64y	78/3705 (2.1%)	43/2224 (1.9%)	1/76 (1.3%)	1/28 (3.6%)	0/45 (0%)	1/43 (2.3%)	1/11 (9.1%)
65-74y	170/2255 (7.5%)	93/1533 (6.1%)	1/48 (2.1%)	1/21 (4.8%)	0/27 (0%)	1/38 (2.6%)	0/3 (0%)
≥ 75y	752/5485 (13.7%)	358/3149 (11.4%)	9/127 (7.1%)	7/60 (11.7%)	2/65 (3.1%)	3/68 (4.4%)	5/26 (19.2%)

*aLRTD* acute lower respiratory tract disease, *CRDE* Chronic respiratory disease exacerbation, *HF* Heart failure, *ICU* Intensive care unit, *IQR* Interquartile range, *NP-LRTI* Non-pneumonic lower respiratory tract infection, *N* Number, *RSV* Respiratory syncytial virus *y* years

**Fig. 3:**
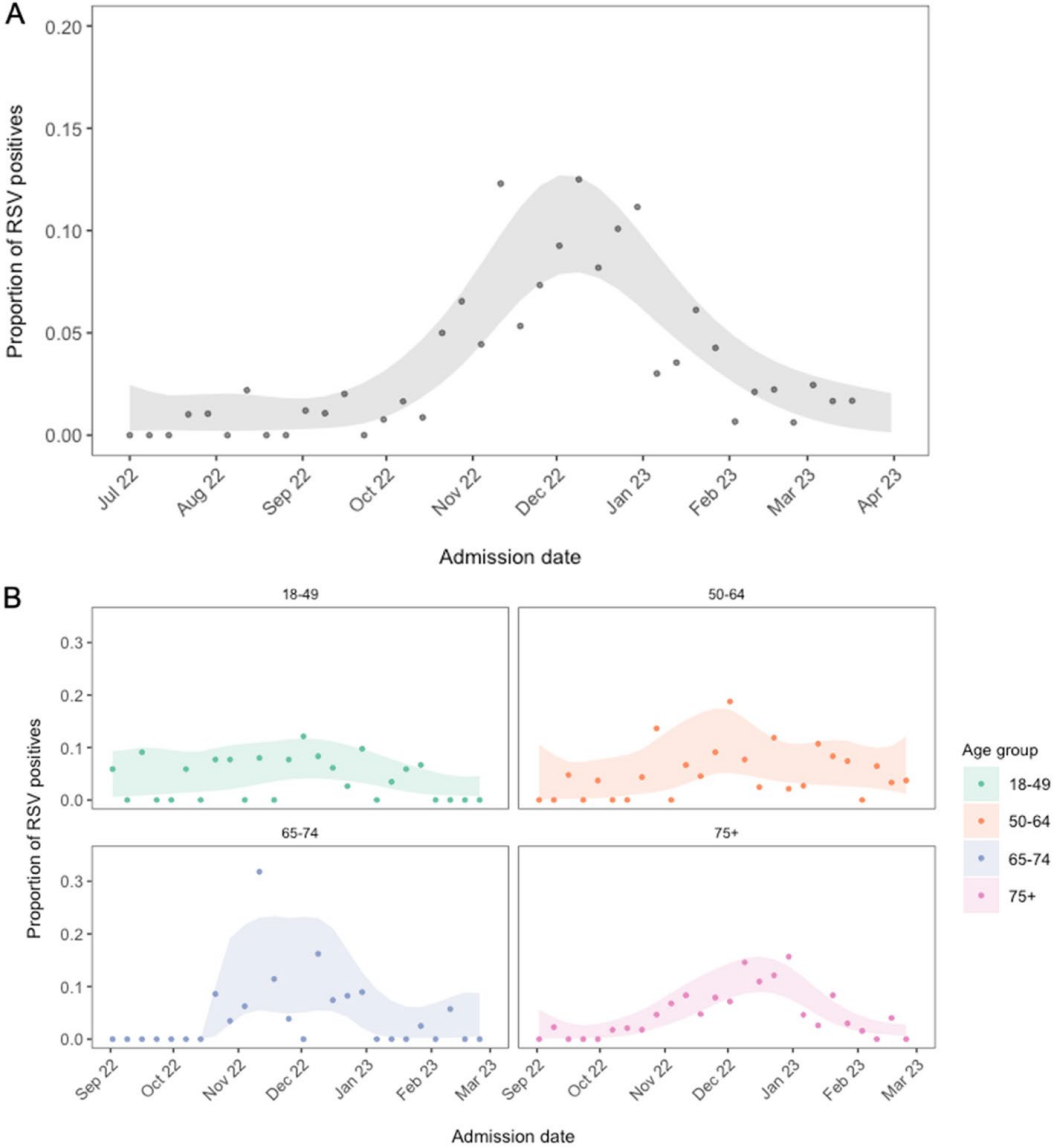
RSV test-positivity over time. The proportion of RSV test-positives over time, overall between July – April (**A**) and stratified by age group between September—March (**B**). Points show observed test-positive-rates for each week (participants RSV test-positive/participants RSV tested), and the ribbons show estimated uncertainty from a locally fitted quasi-binomial model

The overall RSV test-positive-rate in hospitalised adults was 4.4% (95%CI:3.9–5.0%), based on SOC testing, and 3.6% (3.2–4.1) when research testing was added (Table 1). The overall RSV test-positive-rate trended towards increasing with patient age, and across all age groups we found more RSV test-positive individuals in the NP-LRTI subgroup than in the pneumonia subgroup (Table 2). The overall RSV test-positivity rate showed a seasonal pattern, with modelled rates peaking in December between 7.9–12.7% (Fig. 3a). When stratified by age, the modelled test-positive seasonal peak was most clearly seen, and was latest, in the oldest age group (≥ 75y; Fig. 3b). During the off-peak season the SOC test-positivity was 1.4% (1.0–1.9), and the research lab test-positivity was 0.5% (0.2–0.9).

### Adjustment for test error

The latent class (LC) model performed well on simulated data with known parameters, where test sensitivities and specificities were assumed to be the same (Supplementary, Data 1;2), when test sensitivities and specificities varied (Supplementary, Data 3), and when dependence was modelled between the research tests (Supplementary, Data 4). Model inferences were not sensitive to changes in the prior distribution for specificity (Supplementary, Data 5). Small differences between inferred and simulated sensitivity did not impact inferred true prevalence.

**Fig. 4:**
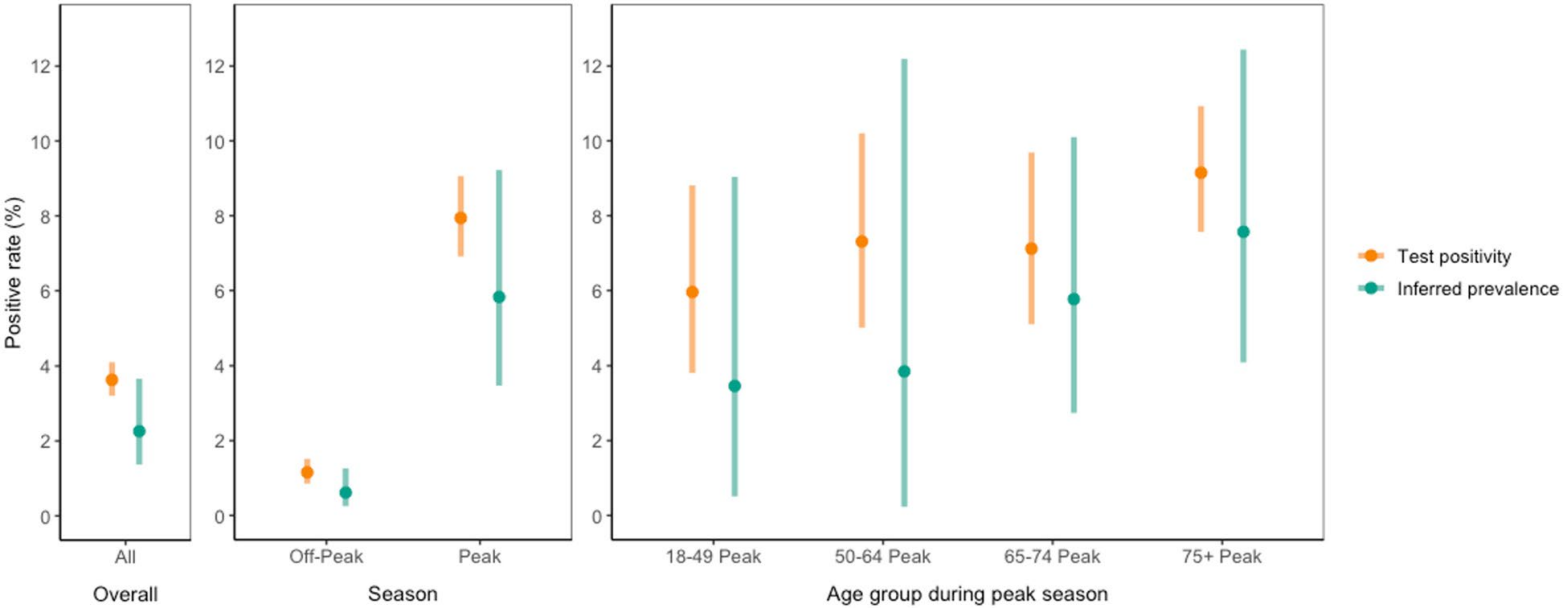
Test-positivity vs inferred true prevalence in different participant subgroups. Point estimates of test-positivity (individuals positive by any test/individuals tested; orange) and inferred true prevalence from a latent class model accounting for test error and multiple testing (mean; green), run for different participant subgroups split by peak season (Nov-Feb), off-peak season, and age groups during the peak season. Bars show 95% CI’s (orange: Clopper and Pearson confidence intervals; green: credible interval)

The LC model inferred a true prevalence of 2.3% (95%CI:1.4–3.7%) in the tested population, compared with a test-positivity of 3.6% (3.2–4.1; 251/6906). Test-positivity overestimated inferred true prevalence in all age- and season-stratified subgroups (Fig. 4). The LC model for the peak RSV season (Nov-Feb) inferred a true prevalence of 5.8% (3.5–9.2), compared to test-positivity of 7.9% (6.9–9.1). The model inferred a higher sensitivity and lower specificity for the SOC test and the sputum research lab sample compared to the NP/OP and saliva research lab samples (Table 4). These trends were common across subgroups, although uncertainty in test sensitivity was large (Extended Data, 7). The model inferred similar estimates of true prevalence, sensitivity and specificity, overall, and for different participant subgroups, when SOC testing was split into the three different panel tests used, and when only the first SOC RSV test was included (Supplementary, Data 6;7).

**Table 4:** Latent class inferred test sensitivities and specificities. Sensitivity and specificity median inferences from study data (*N* = 6906) of 4 different tests and sample types for RSV (Standard of care (SOC NP/OP); and three research lab tests: (NP/OP; saliva, and sputum)) from a Bayesian latent class model (mean across chains). The model includes a dependence structure between the three research lab samples. 95% credible intervals are shown. The LC model inferred a true prevalence of 2.3% (95%CI 1.4–3.7%)

Test and sample type	Sensitivity (95% CI)	Specificity (95% CI)
SOC NP/OP	0.864 (0.677–0.974)	0.984 (0.979–0.991)
Lab NP/OP	0.468 (0.171–0.752)	0.999 (0.998–1.00)
Lab Saliva	0.452 (0.147–0.757)	0.999 (0.997–1.00)
Lab sputum	0.865 (0.569–0.995)	0.993 (0.983–0.999)

**Fig. 5:**
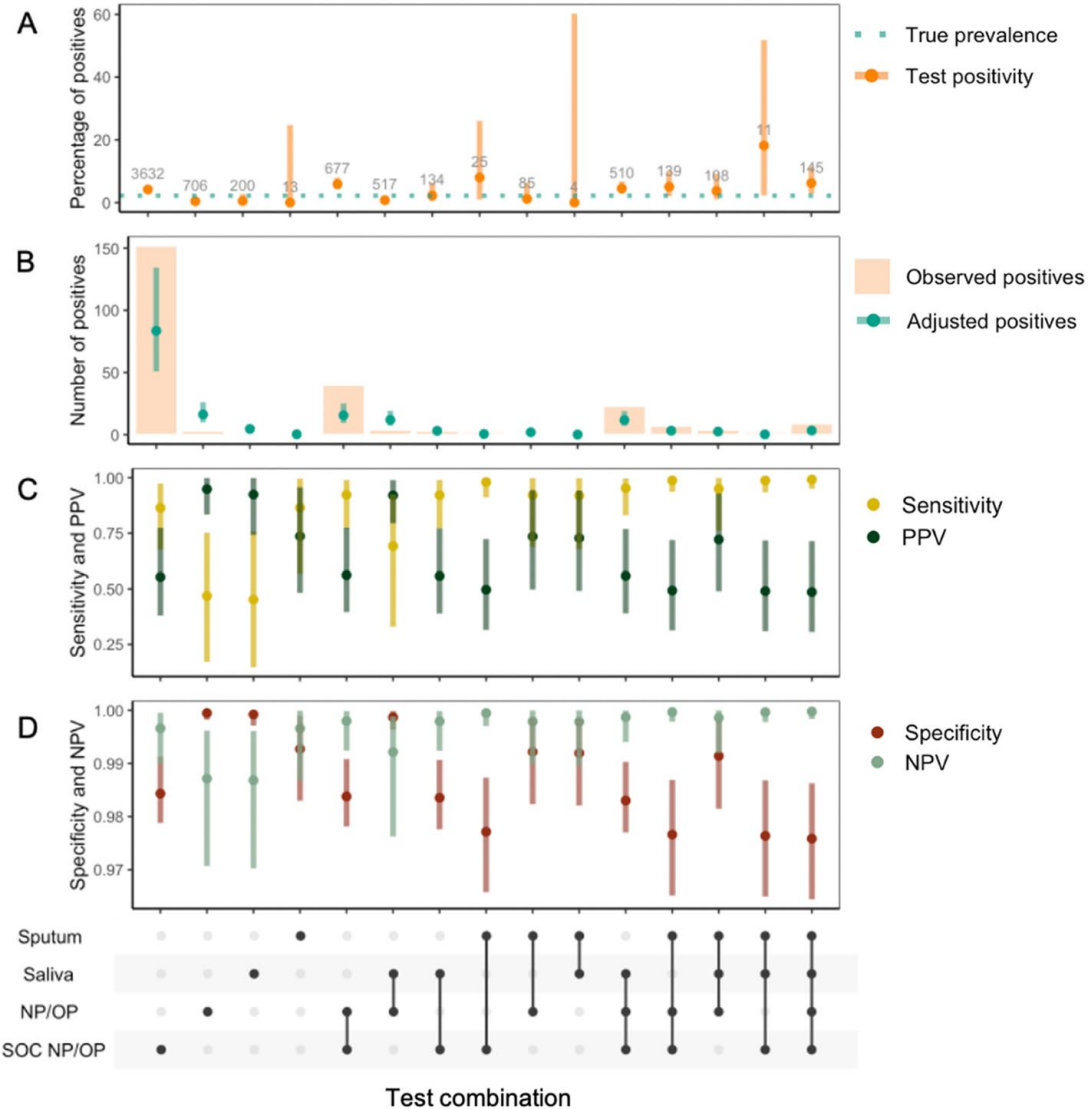
Test characteristics by different combinations of respiratory specimens. UpSet diagrams showing individuals tested by different mutually exclusive test/sample combinations of standard of care (SOC) naso- or oropharyngeal (NP/OP) respiratory swabs and research samples: sputum, saliva or NP/OP respiratory swabs. **A** Percentage of RSV test-positive individuals (95%CI Clopper-Pearson). Numbers show number of participants tested. Dotted line shows true prevalence inferred from a latent class (LC) model; **B** Number of RSV test-positive individuals. Points show number of RSV-positives after adjustment for true prevalence inferred from the LC model (with 95% credible intervals); **C** Sensitivity and positive predictive values (PPV); D) Specificity and negative predictive values (NPV) from LC model (with 95% credible intervals)

At the low test-positivity rates observed in this study, test sensitivity had little impact on accuracy of prevalence estimations, and overestimation of true prevalence was driven by imperfect test specificity (Extended Data, 8; Fig. 5). A Rogan-Gladen estimator [48] demonstrated, at the inferred sensitivity and specificity of the SOC test, test-positivity would not be an underestimation of true prevalence unless test-positivity was > 15% (Extended Data, 9). Therefore, combining highly specific research lab samples with SOC testing, resulted in a lower combined test specificity and PPV (Fig. 5).

### RSV incidence

After adjustment of test-positivity for test error, and multiple testing, and accounting for the untested population, the overall calculated adult population RSV incidence/1000-person-years was 0.33 (95%CI:0.21–0.49). This ranged from 0.06 (0.0–0.12) in the youngest age group (18-49y), to 2.02 (1.10–3.06) in the oldest age group (≥ 75y; Table 5).

**Table 5:** Annual incidence of aLRTD hospitalised patients and RSV/1000-person years. Incidence of aLRTD hospitalisation and hospitalised RSV per 1000-person-years (PYs), stratified by age group. RSV incidence is based on inferred true prevalence of RSV from latent class models which adjust for test error and multiple testing. Latent class models were run separately for each age group for the peak RSV season, and an overall model was run for the off-peak season. The number of positives in each age group is the number of positives in the peak and off-peak season combined (see methods for details). 95% CIs are shown in brackets (95% credible intervals for the combined distribution of number of positives for each age group)

		**All included aLTRD hospitilised**					**RSV hospitilised**
		**Total (all-cause)**	**Pneumonia**	**NP-LRTI**	**CRDE**	**HF**	**Total**
**Age**	**Population**	**per 1000 PYs**	**per 1000 PYs**	**per 1000 PYs**	**per 1000 PYs**	**per 1000 PYs**	**per 1000 PYs**
18–49	435,136	4.10	1.30	2.30	1.80	0.20	0.06 (0.02—0.12)
50–64	157,846	12.20	4.90	5.60	6.50	1.50	0.16 (0.03—0.41)
65–74	72,997	30.90	13.50	12.60	17.50	5.60	0.64 (0.34—1.04)
≥ 75	70,805	77.50	36.90	30.60	32.20	19.00	2.02 (1.10—3.06)
Overall	736,784	15.50	6.70	6.70	7.30	2.80	0.33 (0.21—0.49)

## Discussion

This prospective study found 251/6906 (3.6%; 95%CI:3.2–4.1%) of adults hospitalised with community-acquired aLRTD and tested for RSV in Bristol, UK, were test-positive. We modelled a weekly maximum test-positivity of 7.9–12.7%, with the seasonal peak more prominent in older adults. Recorded rates of RSV test-positivity generally increased with patient age, and a higher RSV test-positive-rate was found among NP-LRTI than pneumonia cases. Over the 12-month study period, 4.2% of CRDE cases and 3.2% of HF cases, that were tested for RSV, were RSV test-positive.

The epidemiology of RSV was disrupted by social distancing measures implemented to reduce infections and hospitalisations following SARS-CoV-2 emergence, with subsequent out-of-season peaks, probably caused by waning population immunity [49–51]. Our analysis is the first UK study to report the RSV-positive-rate following SARS-CoV-2 emergence, and in adults hospitalised with ARI across aLRTD and its subgroups. We show that between April 2022-March 2023, the RSV test-positive peak in adults had returned to the standard winter season, peaking between Nov–Feb, consistent with trends observed across 2022/23 by UKHSA sentinel surveillance [51]. The maximum modelled weekly RSV test-positivity of 7.9–12.7% in all adults is comparable to the 13.7% maximum reported SARS-CoV-2 positive-rate in 65-year-olds in 2022–23 in the UK, but lower than the 26.1% recorded for influenza in ≥ 65-year-olds [51]. Our incidence estimates’ confidence intervals (0.34–1.04 [65-74y]; 1.10–3.06 [≥ 75y]/1000 population) encompass other modelled estimates for the UK, with estimated RSV hospitalisation incidence/1000 population of 0.77 (0.46–1.09) [21] in 65–74-year-olds, and between 1.16 (0.91–1.48) and 3.38 (2.50–4.05) for low and high risk adult populations, respectively, in > 75-year-olds (with risk populations based on chronic conditions indicative of severe influenza risk) [13]. Our incidence estimates fall within these low and high risk adult population estimates for all age groups [13]. Our estimates are comparable to other European countries, with overall modelled hospitalisation incidence/1000 population of 0.66 (0.55–0.76) in 65–75-year-olds [21], and to other high-income countries worldwide, with a pooled meta-analysis estimating 0.9–2.2 hospitalisation incidence/1000 population in > 60-year-olds [52]. This study adds to the small number of prospective studies quantifying adult RSV burden outside of the US [53].

The LCA performed well in inferring both true prevalence and test error parameters. Overall test-positivity is overwhelmingly affected by test specificity in our study. Test sensitivity had very little impact on overall test-positivity. At the parameter ranges in this study, overall test-positivity always overestimated true prevalence (Extended Data, 8). This is expected when the disease of interest is uncommon, as underlying false positive-rates, even if they are low, have a high relative effect on the true positive-rate [22]. This effect will be accentuated when multiple tests are done if all positive results are assumed to be true positives, as this increases overall sensitivity at the cost of overall specificity [25], resulting in a lower positive predictive value of the combination of multiple tests than the individual tests. The PPV will be particularly low in studies, such as this one, that include out-of-season testing for RSV when infection rates are especially low [48]. In our study, most RSV-positive individuals were detected by SOC testing (232/251), therefore it is expected that imperfect SOC specificity (inferred to be 98.4%), will result in a significant proportion of test-positives being false positives (Fig. 5). Thus, even in the absence of RSV in the population, we would expect to see a test-positivity rate of 1.6% representing test noise. Consistent with this, in our off-season population, the SOC positivity-rate was 1.4%. As RSV incidence in adults is generally low [21, 51, 53], adjustment of test-positivity for both the false negative and false positive-rate, as demonstrated here, is important for accurate estimates of adult RSV burden in studies worldwide.

Factors other than test error will also affect accurate ascertainment of true population prevalence. Studies only investigating pneumonia will exclude cases found in NP-LRTI, HF and CRDE, and we note the RSV positivity-rate was higher in NP-LRTI than pneumonia-LRTI and confirm that RSV is associated with CRDE and HF. Comorbid disease, and hence the risk posed by RSV infection [54] accumulates with patient age; however, older patients are less likely to undergo SOC testing [17]. This may disproportionately under-estimate RSV burden among the patient groups most at risk. In this study, research samples were taken an average of 2-days later than SOC samples (Extended Data, 5), so RSV titres in research samples would be predicted to be lower, contributing to the low inferred sensitivities of these tests. However, the LC approach infers sensitivities/specificities of the end-to-end testing system (as opposed to laboratory test sensitivities/specificities), accounting for differences in pre-test probability caused by different participant selection criteria for testing, sampling errors, and the time between sampling and laboratory handling.

The lower sensitivity of the research test in our study means adding sputum or saliva sample testing to NP/OP samples increased apparent RSV detection. This aligns with previous studies that found adding both saliva and sputum sample results increased apparent RSV detection by 63% over NP results alone [19], while adding sputum or saliva test results alone increased apparent detection by 39–52% [18, 19, 55] and 55% [19], respectively, assuming perfect test specificity [19]. Of practical relevance, all these specimens may be obtained during acute illness and thus results may inform patient management, whereas serology requires a convalescent sample. Saliva can be a reliable and sensitive specimen type for RSV and other respiratory viruses [19, 56]. Our research lab NP/OP and saliva samples both had perfect specificity, so combining these results would increase true RSV detection (Fig. 5). We found the sensitivity of sputum samples was ~ twice as high as for NP/OP swabs. In older adults, sputum has stronger RSV [57, 58], influenza [59], and coronavirus [59, 60] PCR signals than nasal swabs. Taken together, these results suggest that future studies relying solely on low sensitivity and high specificity tests, could achieve more accurate estimates of RSV disease burden by testing multiple specimen types. However, if any test has imperfect specificity, such multiple testing will amplify this and result in overestimation of true RSV prevalence, unless adjusted for in the way we demonstrate here.

Our study has many strengths, including the comprehensive ascertainment of LRTI in adults hospitalised within a defined geographical area. Notably, we undertook case-by-case ascertainment, the epidemiological gold-standard [61], and did not rely on clinical coding, a microbiological database or modelling alone to estimate RSV disease. Inclusion of patient data obtained under Sect. 251 of the 2006 NHS Act allows us to make accurate comparisons and draw appropriate conclusions about the representativeness of our sample. We enrolled adults who lacked capacity through a consultee, undertaking enhanced research sampling and avoiding under-representing these patients in this study, including those with aLRTD who are severely ill or have advanced dementia or other frailty. Finally, incidence was calculated using a population denominator derived from healthcare utilisation data, providing increased accuracy compared to estimates based on assumptions using local geographic boundaries and their corresponding census data [47].

The study also has some limitations. Despite undertaking research testing to increase the proportion tested, 40% of the cohort were not tested for RSV. However, the proportion of untested participants was lower (26%) during the peak season (Nov-Feb), in part due to increased clinical suspicion resulting in increased SOC testing during this period. We found statistically significant differences between RSV tested and untested patients, and therefore the underlying true prevalence in these two groups is likely to differ due to differing patient characteristics. For population incidence calculations, inferred true prevalence from the LC models were assumed to be the same in the equivalent untested population subgroup. Stratifying by age group and season controls for the biggest factors affecting clinical suspicion and pre-test probability, especially as RSV is characterised by non-specific respiratory symptoms. Nevertheless, without data on the rationale behind testing decisions, more precise differences between the underlying true prevalence in the tested and untested population cannot be adjusted for. Finally, this study was a single-centre study, and while the Bristol population is representative of the UK, it is predominantly White-British (84% [62]) and therefore aLRTD, RSV disease and RSV testing practices in other cohorts may vary from those reported here.

In conclusion, this prospective study is the first to estimate the RSV burden among adults hospitalised with aLRTD and its subgroups in the UK post-COVID. We found that 3.6% (95%CI:3.2–4.1) of annual RSV tested aLRTD hospital cases were RSV test-positive, with modelled weekly test-positivity peaking at 7.9–12.6%. Overall, test-positivity rates increased with patient age, were associated with a 4.4% all-cause 30-day mortality among hospitalised adults, and 3.6% of adults hospitalised with an RSV-positive test required ICU-level care. Test-positivity (3.6%) overestimated inferred true prevalence (2.3%), due to the low positivity-rates and imperfect test specificity, which was exacerbated by out-of-season and multiple testing. After LC adjustment for test characteristics, we estimated overall adult population incidence/1000-person-years to be 0.33 (0.21–0.49), and 2.02 (1.10–3.06) in ≥ 75-year-olds. The implementation of new RSV vaccines has the potential to reduce the burden of RSV-associated hospitalisation in older adults, especially among the elderly, and the accuracy of future studies of adult RSV burden and vaccine-effectiveness can be improved by adjustment for error introduced by test characteristics.

## Supplementary Information

Supplementary Material 1.

## Data Availability

The data used in this study are sensitive and cannot be made publicly available without breaching patient confidentiality rules. Therefore, individual participant data and a data dictionary are not available to other researchers. For further details about the raw data used in this study and its availability please contact Catherine Hyams (Catherine.Hyams@bristol.ac.uk).

## References

[1] Langley GF, Anderson LJ. Epidemiology and prevention of respiratory syncytial virus infections among infants and young children. Pediatr Infect Dis J. 2011. 10.1097/INF.0b013e3182184ae7. 21487331 10.1097/INF.0b013e3182184ae7

[2] Branche AR, Falsey AR. Respiratory syncytial virus infection in older adults: an under-recognized problem. Drugs Aging. 2015. 10.1007/s40266-015-0258-9. 25851217 10.1007/s40266-015-0258-9

[3] Branche AR, Saiman L, Walsh EE, Falsey AR, Sieling WD, Greendyke W, et al. Incidence of respiratory syncytial virus infection among hospitalized adults, 2017–2020. Clin Infect Dis. 2022. 10.1093/cid/ciab595. 34244735 10.1093/cid/ciab595

[4] Nguyen-Van-tam JS, O’leary M, Martin ET, Heijnen E, Callendret B, Fleischhackl R, Comeaux C, Tran TMP, Weber K. Burden of respiratory syncytial virus infection in older and high-risk adults: a systematic review and meta-analysis of the evidence from developed countries. Eur Respir Rev. 2022;31(166):220105. 10.1183/16000617.0105-2022. PMID: 36384703; PMCID: PMC9724807. 10.1183/16000617.0105-2022 PMC9724807 36384703

[5] Li Y, Kulkarni D, Begier E, Wahi-Singh P, Wahi-Singh B, Gessner B, et al. Adjusting for case under-ascertainment in estimating RSV hospitalisation burden of older adults in high-income countries: a systematic review and modelling study. Infect Dis Ther. 2023;12(4):1137–49. 36941483 10.1007/s40121-023-00792-3 PMC10027261

[6] Kelleher K, Subramaniam N, Drysdale SB. The recent landscape of RSV vaccine research. Ther Adv Vaccines Immunother. 2025;10: 13. 10.1177/25151355241310601 PMC11724408 39802673

[7] UKSHA, NHS England. Introduction of new NHS vaccination programmes against respiratory syncytial virus (RSV). https://www.gov.uk/government/publications/respiratory-syncytial-virus-rsv-vaccination-programmes-letter/introduction-of-new-nhs-vaccination-programmes-against-respiratory-syncytial-virus-rsv. 2024 Jun.

[8] Walsh EE, Pérez Marc G, Zareba AM, Falsey AR, Jiang Q, Patton M, et al. Efficacy and safety of a bivalent RSV prefusion F vaccine in older adults. N Engl J Med. 2023;388(16):1465–77. 37018468 10.1056/NEJMoa2213836

[9] Falsey AR, Cameron A, Branche AR, Walsh EE. Perturbations in respiratory syncytial virus activity during the SARS-CoV-2 pandemic. J Infect Dis. 2023. 10.1093/infdis/jiac434. 36315855 10.1093/infdis/jiac434

[10] Jefferson T, Del Mar CB, Dooley L, Ferroni E, Al-Ansary LA, Bawazeer GA, et al. Physical interventions to interrupt or reduce the spread of respiratory viruses. Cochrane Database Syst Rev. 2011;2020. 10.1002/14651858.CD006207.pub5. 10.1002/14651858.CD006207.pub3 20091588

[11] Brooks-Pollock E, Read JM, McLean AR, Keeling MJ, Danon L. Mapping social distancing measures to the reproduction number for COVID-19. Philos Trans R Soc B Biol Sci. 2021;376(1829). 10.1098/rstb.2020.0276. 10.1098/rstb.2020.0276 PMC8165600 34053268

[12] Choi YH, Miller E. Impact of COVID-19 social distancing measures on future incidence of invasive pneumococcal disease in England and Wales: a mathematical modelling study. BMJ Open. 2021. 10.1136/bmjopen-2020-045380. 34588227 10.1136/bmjopen-2020-045380 PMC8479589

[13] Fleming DM, Taylor RJ, Lustig RL, Schuck-Paim C, Haguinet F, Webb DJ, et al. Modelling estimates of the burden of Respiratory Syncytial virus infection in adults and the elderly in the United Kingdom. 2015;15(1). 10.1186/s12879-015-1218-z. 10.1186/s12879-015-1218-z PMC4618996 26497750

[14] Sharp A, Minaji M, Panagiotopoulos N, Reeves R, Charlett A, Pebody R. Estimating the burden of adult hospital admissions due to RSV and other respiratory pathogens in England. Influenza Other Respi Viruses. 2022;16(1):125–31. 10.1111/irv.12910 PMC8692807 34658161

[15] Johannesen CK, van Wijhe M, Tong S, Fernández LV, Heikkinen T, van Boven M, et al. Age-specific estimates of respiratory syncytial virus-associated hospitalizations in 6 European Countries: a time series analysis. J Infect Dis. 2022;226(Supplement_1):S29-37. 35748871 10.1093/infdis/jiac150

[16] Rozenbaum MH, Judy J, Tran D, Yacisin K, Kurosky SK, Begier E. Low levels of RSV testing among adults hospitalized for lower respiratory tract infection in the United States. Infect Dis Ther. 2023. 10.1007/s40121-023-00758-5. 36707466 10.1007/s40121-023-00758-5 PMC9883084

[17] Hyams C, Begier E, Garcia Gonzalez M, Southern J, Campling J, Gray S, et al. Incidence of acute lower respiratory tract disease hospitalisations, including pneumonia, among adults in Bristol, UK, 2019, estimated using both a prospective and retrospective methodology. BMJ Open. 2022. 10.1136/bmjopen-2021-057464. 35705333 10.1136/bmjopen-2021-057464 PMC9204403

[18] Onwuchekwa C, Moreo LM, Menon S, Machado B, Curcio D, Kalina W, et al. Underascertainment of respiratory syncytial virus infection in adults due to diagnostic testing limitations: a systematic literature review and meta-analysis. J Infect Dis. 2023;228(2):173–84. 36661222 10.1093/infdis/jiad012 PMC10345483

[19] Ramirez J, Carrico R, Wilde A, Junkins A, Furmanek S, Chandler T, et al. Diagnosis of respiratory syncytial virus in adults substantially increases when adding sputum, saliva, and serology testing to nasopharyngeal swab RT–PCR. Infect Dis Ther. 2023. 10.1007/s40121-023-00805-1. 37148463 10.1007/s40121-023-00805-1 PMC10163290

[20] Rozenbaum MH, Begier E, Kurosky SK, Whelan J, Bem D, Pouwels KB, et al. Incidence of respiratory syncytial virus infection in older adults: limitations of current data. Infect Dis Ther. 2023;12:1487–504. 37310617 10.1007/s40121-023-00802-4 PMC10262134

[21] Osei-Yeboah R, Spreeuwenberg P, Del Riccio M, Fischer TK, Egeskov-Cavling AM, Bøås H, et al. Estimation of the number of respiratory syncytial virus-associated hospitalizations in adults in the European Union. J Infect Dis. 2023;228(11):1539–48. 37246742 10.1093/infdis/jiad189 PMC10681866

[22] Gelman A, Carpenter B. Bayesian analysis of tests with unknown specificity and sensitivity. J Royal Stat Soc Ser C: Appl Stat. 2020. 10.1111/rssc.12435. 10.1111/rssc.12435 PMC10016948 37252679

[23] Parikh R, Mathai A, Parikh S, Chandra Sekhar G, Thomas R. Understanding and using sensitivity, specificity and predictive values. 2008. 10.4103/0301-4738.37595. 10.4103/0301-4738.37595 PMC2636062 18158403

[24] Maxim LD, Niebo R, Utell MJ. Screening tests: a review with examples. Inhal Toxicol. 2014;26(13):811–28. 25264934 10.3109/08958378.2014.955932 PMC4389712

[25] Zhou X, McClish D, Obuchowski N. Statistical methods in diagnostic medicine. 1st ed. Wiley 2002. 50–51.

[26] Dawid AP, Skene AM. Maximum likelihood estimation of observer error-rates using the EM Algorithm. J R Stat Soc Ser C Appl Stat. 1979;28. 10.2307/2346806.

[27] Hui SL, Walter SD. Estimating the error rates of diagnostic tests. Biometrics. 1980. 10.2307/2530508. 7370371

[28] van Smeden M, Naaktgeboren CA, Reitsma JB, Moons KGM, de Groot JAH. Latent class models in diagnostic studies when there is no reference standard–a systematic review. Am J Epidemiol. 2014;179(4):423–31. 24272278 10.1093/aje/kwt286

[29] Ferraz MB, Walter SD, Heymann R, Atra E. Sensitivity and specificity of different diagnostic criteria for behcet’s disease according to the latent class approach. Br J Rheumatol. 1995. 10.1093/rheumatology/34.10.932. 7582698 10.1093/rheumatology/34.10.932

[30] Hadgu A, Qu Y. A biomedical application latent class models with random effects. J R Stat Soc Ser C Appl Stat. 1998;47. 10.1111/1467-9876.00131.

[31] Moayyedi P, Duffy J, Delaney B. New approaches to enhance the accuracy of the diagnosis of reflux disease. Gut. 2004;53. 10.1136/gut.2003.034363. 10.1136/gut.2003.034363 PMC1867783 15082616

[32] Chappuis F, Rijal S, Jha UK, Desjeux P, Karki BMS, Koirala S, et al. Field validity, reproducibility and feasibility of diagnostic tests for visceral leishmaniasis in rural Nepal. Trop Med Int Heal. 2006;11(1):31–40. 10.1111/j.1365-3156.2005.01533.x 16398753

[33] MacLean EL, Kohli M, Köppel L, Schiller I, Sharma SK, Pai M, et al. Bayesian latent class analysis produced diagnostic accuracy estimates that were more interpretable than composite reference standards for extrapulmonary tuberculosis tests. Diagnostic Progn Res. 2022;6(1). 10.1186/s41512-022-00125-x. 10.1186/s41512-022-00125-x PMC9202094 35706064

[34] Challen R, Chatzilena A, Qian G, Oben G, Kwiatkowska R, Hyams C, et al. Combined multiplex panel test results are a poor estimate of disease prevalence without adjustment for test error. PLoS Comput Biol. 2024. 10.1371/journal.pcbi.1012062. 38669293 10.1371/journal.pcbi.1012062 PMC11078360

[35] Hyams C, Challen R, Begier E, Southern J, King J, Morley A, et al. Incidence of community acquired lower respiratory tract disease in Bristol, UK during the COVID-19 pandemic: a prospective cohort study. Lancet Reg Heal - Eur. 2022;21:100473. 10.1016/j.lanepe.2022.100473 PMC9359590 35965672

[36] Harris PA, Taylor R, Thielke R, Payne J, Gonzalez N, Conde JG. Research electronic data capture (REDCap)-a metadata-driven methodology and workflow process for providing translational research informatics support. J Biomed Inform. 2009. 10.1016/j.jbi.2008.08.010. 18929686 10.1016/j.jbi.2008.08.010 PMC2700030

[37] Charlson ME, Pompei P, Ales KL, MacKenzie CR. A new method of classifying prognostic comorbidity in longitudinal studies: development and validation. J Chronic Dis. 1987. 10.1016/0021-9681(87)90171-8. 3558716 10.1016/0021-9681(87)90171-8

[38] Rockwood K, Song X, MacKnight C, Bergman H, Hogan DB, McDowell I, et al. A global clinical measure of fitness and frailty in elderly people. C Can Med Assoc J. 2005;173(5). 10.1503/cmaj.050051. 10.1503/cmaj.050051 PMC1188185 16129869

[39] Lim WS, Van Der Eerden MM, Laing R, Boersma WG, Karalus N, Town GI, et al. Defining community acquired pneumonia severity on presentation to hospital: an international derivation and validation study. Thorax. 2003. 10.1136/thorax.58.5.377. 12728155 10.1136/thorax.58.5.377 PMC1746657

[40] National Institute for Clinical Excellence. Pneumonia in adults: diagnosis and management Clinical guideline. 2023. 31940163

[41] Loader C. Local regression and likelihood. New York: Springer-Verlag; 1999.

[42] R Core Team. R: a language and environment for statistical computing. Vienna, Austria: R Foundation for Statistical Computing; 2021.

[43] Stan Development Team. RStan: the R interface to Stan. 2024.

[44] Qu Y, Tan M, Kutner MH. Random effects models in latent class analysis for evaluating accuracy of diagnostic tests. Biometrics. 1996. 10.2307/2533043. 8805757

[45] Keddie SH, Baerenbold O, Keogh RH, Bradley J. Estimating sensitivity and specificity of diagnostic tests using latent class models that account for conditional dependence between tests: a simulation study. BMC Med Res Methodol. 2023. 10.1186/s12874-023-01873-0. 36894883 10.1186/s12874-023-01873-0 PMC9999546

[46] Makowski D, Ben-Shachar M, Lüdecke D. bayestestR: Describing effects and their uncertainty, existence and significance within the bayesian framework. J Open Source Softw. 2019;4(40). https://joss.theoj.org/papers/10.21105/joss.01541.

[47] Campling J, Begier E, Vyse A, et al. A novel approach to estimate the local population denominator to calculate disease incidence for hospital-based health events in England. Epidemiol Infect. 2022. 10.1017/S0950268822000917. 35811424 10.1017/S0950268822000917 PMC9386789

[48] Rogan WJ, Gladen B. Estimating prevalence from the results of a screening test. Am J Epidemiol. 1978;107(1):71–6. 623091 10.1093/oxfordjournals.aje.a112510

[49] Löwensteyn YN, Zheng Z, Rave N, Bannier MAGE, Billard MN, Casalegno JS, et al. Year-round RSV transmission in the netherlands following the COVID-19 Pandemic - a prospective nationwide observational and modeling study. medRxiv. 2022. 10.1093/infdis/jiad282. 10.1093/infdis/jiad282 PMC10640768 37477906

[50] Stein RT, Zar HJ. RSV through the COVID-19 pandemic: burden, shifting epidemiology, and implications for the future. Pediatr Pulmonol. 2023;58(6):1631–9. 36811330 10.1002/ppul.26370

[51] UKSHA. National Influenza and COVID-19 surveillance report: Week 45 report (up to week 44 data). 2023.

[52] Savic M, Penders Y, Shi T, Branche A, Pirçon JY. Respiratory syncytial virus disease burden in adults aged 60 years and older in high-income countries: a systematic literature review and meta-analysis. Influenza Other Respi Viruses. 2023. 10.1111/irv.13031. 10.1111/irv.13031 PMC9835463 36369772

[53] Doty B, Ghaswalla P, Bohn RL, Stoszek SK, Panozzo CA. Incidence of RSV in adults: a comprehensive review of observational studies and critical gaps in information. J Infect Dis. 2024. 10.1093/infdis/jiae314. 38934801 10.1093/infdis/jiae314 PMC11646608

[54] Widmer K, Zhu Y, Williams JV, Griffin MR, Edwards KM, Talbot HK. Rates of hospitalizations for respiratory syncytial virus, human metapneumovirus, and influenza virus in older adults. J Infect Dis. 2012. 10.1093/infdis/jis309. 22529314 10.1093/infdis/jis309 PMC3415933

[55] Mclaughlin JM, Khan F, Begier E, Swerdlow DL, Jodar L, Falsey AR. Rates of medically attended RSV among US adults: a systematic review and meta-analysis. Open Forum Infect Dis. 2022;9(7):1–10. 10.1093/ofid/ofac300 PMC9301578 35873302

[56] Wyllie AL, Fournier J, Casanovas-Massana A, Campbell M, Tokuyama M, Vijayakumar P, et al. Saliva or nasopharyngeal swab specimens for detection of SARS-CoV-2. N Engl J Med. 2020. 10.1056/NEJMc2016359. 32857487 10.1056/NEJMc2016359 PMC7484747

[57] Walsh EE, Peterson DR, Kalkanoglu AE, Lee FEH, Falsey AR. Viral shedding and immune responses to respiratory syncytial virus infection in older adults. J Infect Dis. 2013;207(9):1424–32. 23382572 10.1093/infdis/jit038 PMC3610422

[58] Branche AR, Walsh EE, Formica MA, Falsey AR. Detection of respiratory viruses in sputum from adults by use of automated multiplex PCR. J Clin Microbiol. 2014;52(10):3590–6. 25056335 10.1128/JCM.01523-14 PMC4187748

[59] Falsey AR, Formica MA, Walsha EE. Yield of sputum for viral detection by reverse transcriptase PCR in adults hospitalized with respiratory illness. J Clin Microbiol. 2012;50(1). 10.1128/JCM.05841-11. 10.1128/JCM.05841-11 PMC3256730 22090400

[60] Jeong JH, Kim KH, Jeong SH, Park JW, Lee SM, Seo YH. Comparison of sputum and nasopharyngeal swabs for detection of respiratory viruses. J Med Virol. 2014. 10.1002/jmv.23937. 24797344 10.1002/jmv.23937 PMC7166652

[61] Honein MA, Paulozzi LJ. Birth defects surveillance: Assessing the “gold standard.” Am J Public Health. 1999. 10.2105/AJPH.89.8.1238. 10432914 10.2105/ajph.89.8.1238 PMC1508690

[62] World Population Review. Bristol Population 2024. https://worldpopulationreview.com/world-cities/bristol-population. 2024.

